# Synthetic Ecosystems: From the Test Tube to the Biosphere

**DOI:** 10.1021/acssynbio.4c00384

**Published:** 2024-11-21

**Authors:** Ricard Solé, Victor Maull, Daniel R. Amor, Jordi Pla Mauri, Conde-Pueyo Núria

**Affiliations:** † ICREA-Complex Systems Lab, Universitat Pompeu Fabra, Dr Aiguader 88, 08003 Barcelona, Spain; ‡ Institut de Biologia Evolutiva, CSIC-UPF, Pg Maritim de la Barceloneta 37, 08003 Barcelona, Spain; ¶ European Centre for Living Technology, Sestiere Dorsoduro, 3911, 30123, Venice, Italy; § Santa Fe Institute, 1399 Hyde Park Road, Santa Fe New Mexico 87501, United States; ∥ LPENS, Département de physique, École normale supérieure, Université PSL, Sorbonne Université, Université Paris Cité, CNRS, 75005 Paris, France; ⊥ IAME, Université de Paris Cité, Université Sorbonne Paris Nord, INSERM, 75005 Paris, France; # EMBL Barcelona, European Molecular Biology Laboratory (EMBL), Barcelona 08003, Spain

**Keywords:** Synthetic Biology, Ecological Engineering, climate change, ecospheres, life support systems

## Abstract

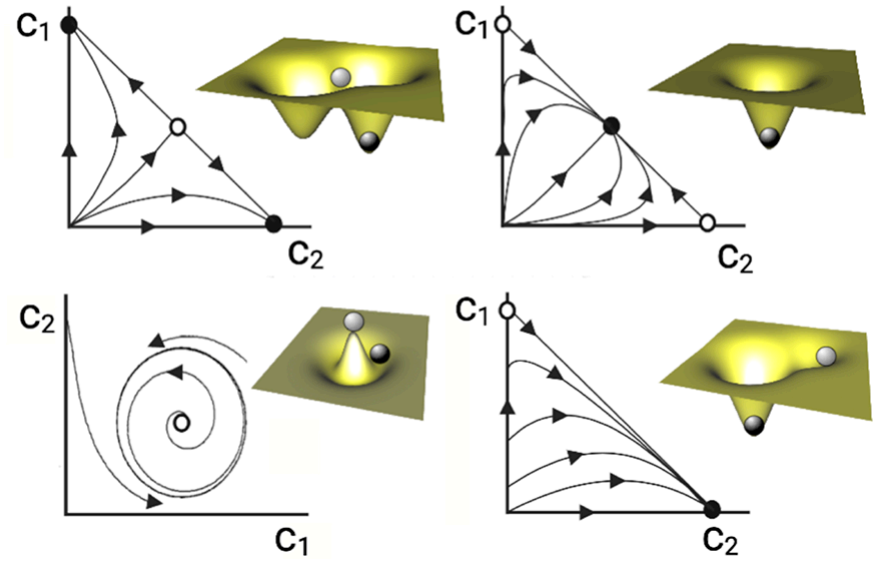

The study of ecosystems, both natural and artificial, has historically been mediated by population dynamics theories. In this framework, quantifying population numbers and related variables (associated with metabolism or biological-environmental interactions) plays a central role in measuring and predicting system-level properties. As we move toward advanced technological engineering of cells and organisms, the possibility of bioengineering ecosystems (from the gut microbiome to wildlands) opens several questions that will require quantitative models to find answers. Here, we present a comprehensive survey of quantitative modeling approaches for managing three kinds of synthetic ecosystems, sharing the presence of engineered strains. These include test tube examples of ecosystems hosting a relatively low number of interacting species, mesoscale closed ecosystems (or ecospheres), and macro-scale, engineered ecosystems. The potential outcomes of synthetic ecosystem designs and their limits will be relevant to different disciplines, including biomedical engineering, astrobiology, space exploration and fighting climate change impacts on endangered ecosystems. We propose a space of possible ecosystems that captures this broad range of scenarios and a tentative roadmap for open problems and further exploration.

## 1 Introduction

**Figure 1:**
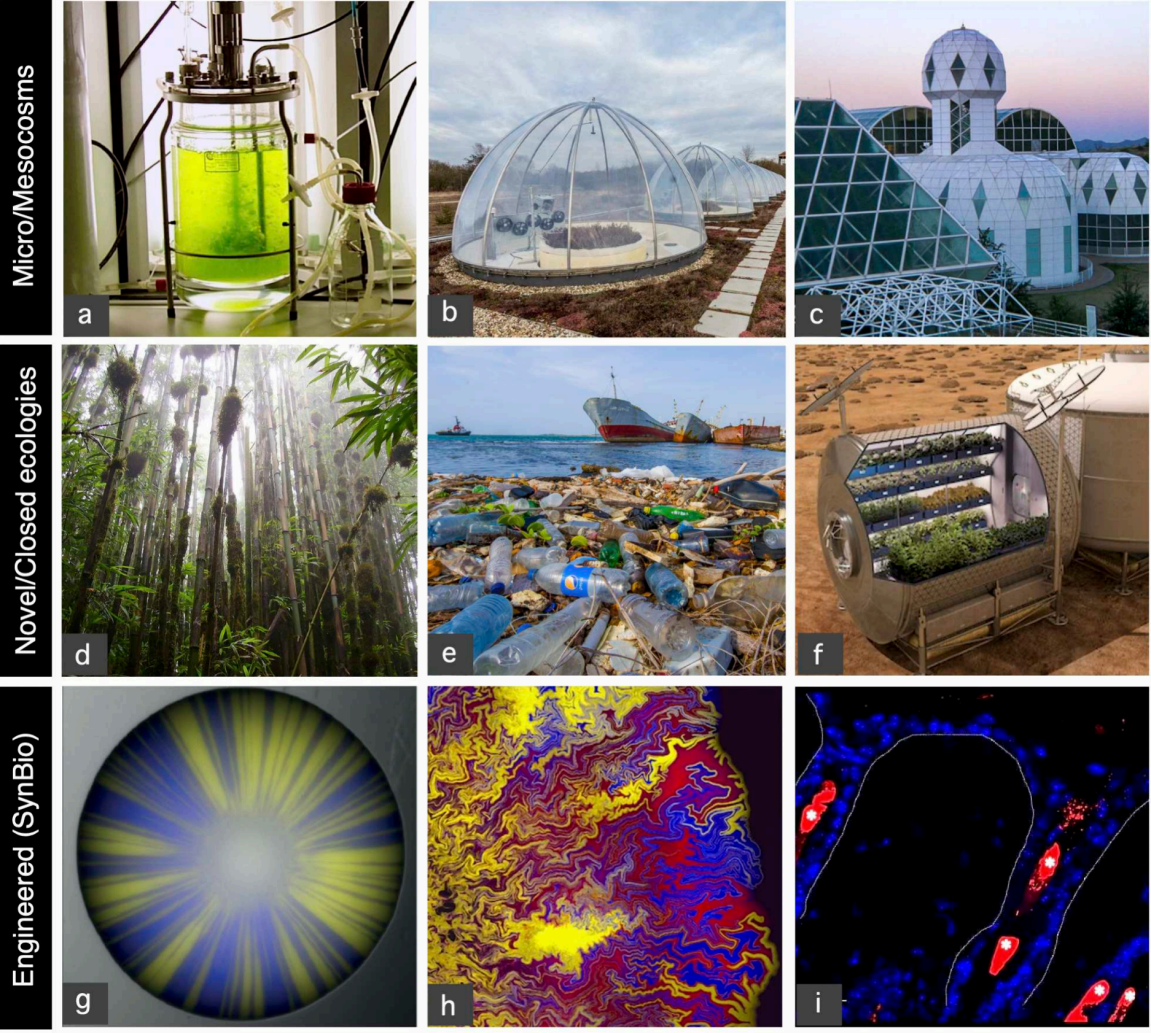
Microcosms, novel, and synthetic ecosystems. Simplified communities have been studied within the context of (a) micro- and (b) mesocosm experiments, while a large-scale scenario is provided by Biosphere 2 (c), including different habitats. New kinds of human-related ecosystems have also been obtained by assembling many exotic species, as is the case of Ascension Island (d), or have emerged within an Anthropogenic waste, such as plastic (e), providing a new habitat to the so-called Plastisphere. Space missions (f) present extra challenges associated with closed environments. Synthetic biology not only offers ways to both interrogate nature, as it occurs with competition (g) and cooperation (h) in the Petri dish, but also as ways to modify extant communities, such as (i) the skin microbiome.

Within ecology, particularly from the early works of Alfred Lotka, population dynamics has been its most relevant quantitative approximation. Populations are naturally described in abundance patterns in time and space, and common properties pervade their organization across scales and population dynamics provides a rationale for understanding how ecosystems work.^1^ Not surprisingly, most textbooks in ecology start their approach to this broad field with simple models that describe the dynamics of a finite number of interacting species and their stability in terms of species abundances. Along with them, it was soon realized that one way to approach ecological complexity could be to construct simpler instantiations of real, much more complex ecosystems, sometimes requiring little more than some initial diversity (Figure 1a). Those so-called *microcosms*(2) enclose small ecosystems held in containers that allow the study of small communities that can be systematically replicated. The experimental tractability of microcosms, together with recent advances in genetic engineering and (meta-)genomics techniques, made them an excellent asset to not only quantitatively testing theoretical predictions for community structure and dynamics,^3−5^ but also assessing how functional performance^6,7^ depends on community composition—i.e. the taxonomic identity of community members.^8^ On an intermediate scale, the same principles were extended into *mesocosms*,^9^Figure 1b and, at least in one case, to very large projects such as Biosphera 2,^10^Figure 1c.

Ecological communities are complex adaptive systems where different scales interact.^11^ Because of their nonlinear nature, they display multiple alternative states, memory effects and breakpoints.^1^ A large body of research has been developed around the population dynamics of microbial ecosystems, where the quest to a predictive understanding is a major issue.^12^ These models have been traditionally instrumental in understanding natural communities, but their role has expanded into other kinds of ecosystems that, in one way or another, result from human interventions. This includes three types outlined in (Figure 1d-f), including (d) Ascension Island, which originated from an artificial combination of exotic species brought to the island,^13^ (e) marine ecosystems that develop on plastic debris or (f) planetary colonization projects involving the building and maintenance of Life Support Systems (LSS, a class of closed ecosystems).

With the rise of synthetic biology at the beginning of the 21st century, a whole new class of ecosystems became a reality: those designed from genetically modified organisms. Because of their designed nature, they can be used to interrogate the laws of ecological complexity. The importance of ecological thinking within synthetic biology is highlighted by the recurrent use of ecosystem-related concepts to understand the population-level behavior of engineered communities.^14^ This is particularly relevant when dealing with the stability and complexity of multicellular consortia, i.e., when different kinds of nonlinear interactions occur among engineered strains or between synthetic and natural cell populations. In Figure 1 (g-h), we provide three examples of them, corresponding to competition between engineered yeast strains^15^ (g) and cooperation between engineered *E. coli* strains^16^ (h) during spatial population expansion on a Petri dish, and (i) engineered *Cutibacterium acnes* strains (in red) colonizing a hair foliculum.^17^ In most of these systems, a full understanding of the (sometimes unexpected) spatiotemporal patterns is achieved using a suitable modeling approach.

There is a last scenario where synthetic ecosystems can play a crucial role: those associated with interventions, either within the domain of bioremediation^18−20^ or as paths toward bioengineering the biosphere.^21−24^ In both cases, a component of genetic design underlying a common goal is to restore or even modify extant ecosystems that have experienced—or can experience—degradation processes or collapse. In these cases, synthetic constructs are deployed in a habitat where a resident community is already present. Consequently, the final community will be ”synthetic” if it incorporates the designed strains, which will coexist (perhaps indefinitely) within its host environment.

In this perspective, we aim to provide a comprehensive overview of various community types categorized as synthetic ecosystems using a population dynamics approach. These ecosystems involve the utilization of engineered strains to establish stable consortia. We situate them within the broader framework of artificial ecosystems to gain insight into their nature and potential applications within three spatial scales. Specifically, we examine three distinct classes of systems: (a) designed synthetic microbial communities, (b) closed ecosystems, and (c) terraformed ecosystems.

The first class encompasses a growing array of designed communities, primarily microbial, crafted through engineered gene networks. These cellular consortia allow us to interrogate the logic of ecological interactions and are the basis for synthetic ecosystem designs. The second class comprises mesoscale engineered closed habitats, i.e., modified to restrict effectively matter flows. Lastly, we explore the potential of large-scale synthetic ecosystems resulting from bioengineering efforts that extend the reach of bioremediation into the restoration of endangered or degraded ecosystems facing potential tipping points. In the three scenarios, systems biology approaches are required to guide our design efforts, and synthetic biology provides a robust path to designing predictable ecosystem architectures across scales.

## 2 Synthetic Microcosms

Microbial communities comprise diverse microorganisms engaging in various interactions, from cross-protection against toxins to resource competition. They harbor the majority of Earth’s genetic diversity and perform essential ecological functions, such as nitrogen fixation and soil erosion prevention. Unlike macro-organism communities, microbial communities can sustain vast populations in small spaces, with billions of bacterial cells proliferating in a single test tube, and exhibit rapid eco-evolutionary dynamics, with generation times of just minutes. Advances in genetic engineering and sequencing have made these communities ideal for testing theoretical predictions and developing synthetic ecosystems.

**Figure 2:**
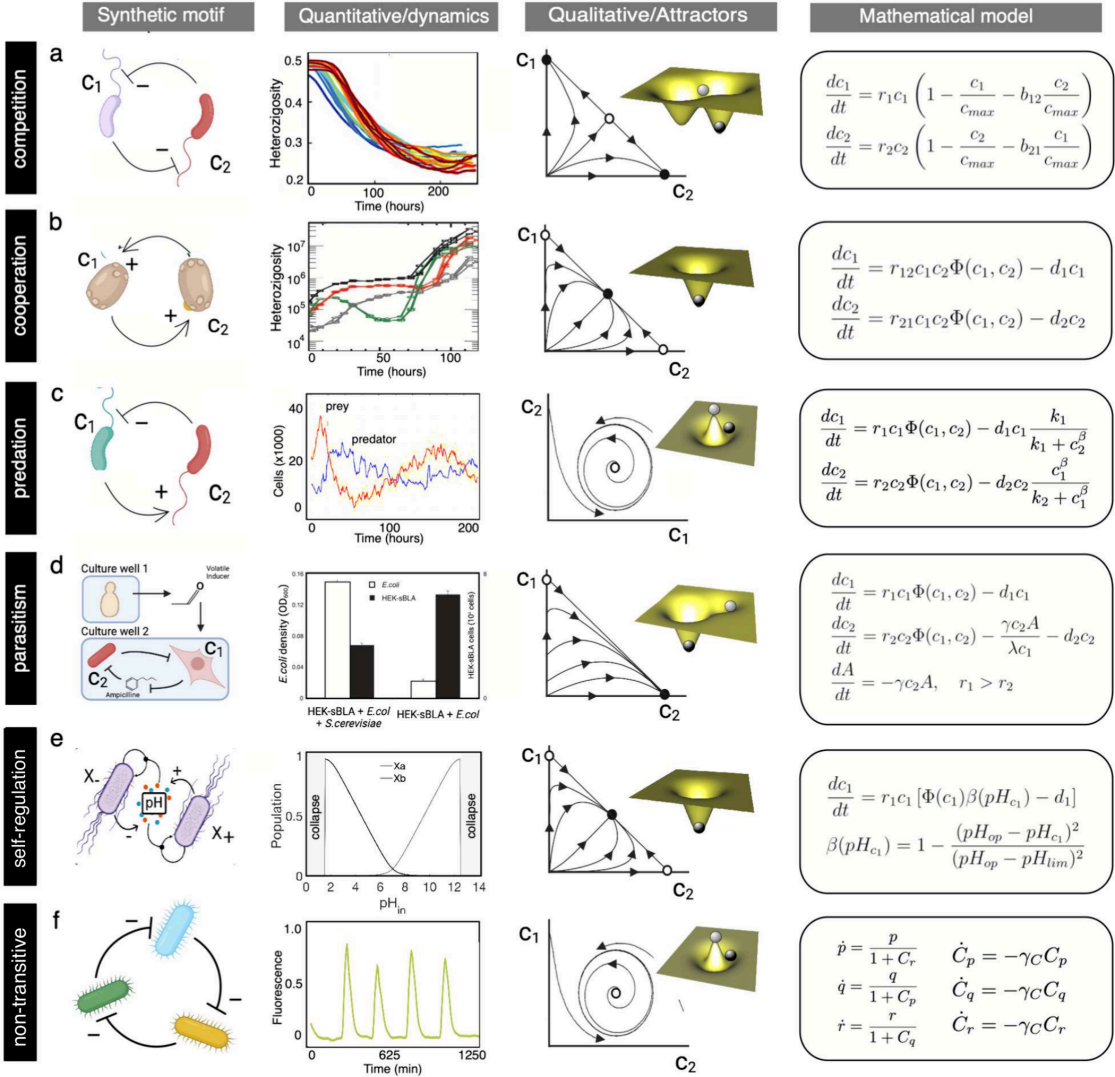
Minimal synthetic ecological networks. Here, we display six types of distinct SE and their characterization using quantitative analyses, qualitative stability properties, and candidate mathematical models. In each case, the nature of the species–species interactions is indicated. At the same time, quantification of their underlying behavior is illustrated by both time series (for the three first examples) and statistical, stationary measures for the last two. Beyond the specific quantitative results, each motif displays some class of generic behavior that can be described in terms of attractor states. Here, we display, on a (*c*_1_, *c*_2_) space, the trajectories exhibited by the system, which connect stable (filled circles) and unstable (open circles) states. In some systems, alternative states are present, while in others there is a single, global attractor and in some they follow a periodic orbit. These attractors are often represented as marbles on a landscape. Here, stable and unstable states (darker and lighter spheres, respectively) correspond to the bottom of valleys and peaks. Simple mathematical models (right column) provide a mechanistic explanation of how nonlinear interactions generate different kinds of attractor states.

Understanding and controlling interactions within microbial communities is essential for managing synthetic microcosms. Microbial interactions impact the growth of target species and are classified as negative, positive, or neutral. These interactions form minimal network motifs that serve as foundational models for engineering complex interaction networks in microbial communities (see Figure 2).

### 2.1 Competition

A classical model for competition between two populations is the competitive Lotka-Volterra (LV) model, which has played a central role in theoretical ecology. In this model, two populations *c*_1_ and *c*_2_ compete for some common implicit resources as follows:
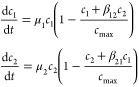
1where μ_*i*_ is the growth rate of population *c_i_* and *c*_max_ is the carrying capacity of the system. The β_*ij*_ parameter is the interspecific competition coefficient. It defines the intensity of competition between the two strains relative to the intraspecific competition coefficient (here normalized β_*ii*_ = 1). Setting all β_*ij*_, *i* ≠ *j*, to zero reduces the system to two independent logistic equations like eq 1.

The competitive LV model displays three qualitative outcomes: coexistence of the two species (only possible when intraspecific competition is stronger than interspecific competition), competitive exclusion (one of the species always leads the other to extinction), and bistability (either species can exclude the other one depending on its initial relative abundance). In the case of exclusion (bistability), one (either) species is subject to an interspecific competition stronger than the intraspecific one, eventually leading to diversity loss as one species reaches extinction (see Figure 2a).

In spatially extended systems, competitive exclusion could be avoided if competition occurs under local dispersal.^25^ One way to visualize the effects of competitive dynamics using engineered strains (see Figure 1g) is to allow an initial inoculum of *P. aeruginosa* YFP- and CFP-labeled mutants.^26^ Because they can interact only with nearest neighbors, a local amplification propagates outward as the colony grows, indicating that each strain overcompetes locally the other.

### 2.2 Cooperation

The presence of cooperative interactions appears to be a driving force behind evolutionary innovations and the preservation of biodiversity. A minimal cooperation scenario involves two strains displaying syntropic dependencies: one species requires the other for replication due to their shared need for a specific molecule produced by their partner species. Synthetic, two-species mutualisms have been engineered in both eukaryotic^27^ and prokaryotic^16,28,29^ systems.

If we indicate by *c*_1_ and *c*_2_ cell population sizes, a pair of coupled equations allows us to model a two-population mutualism as follows:
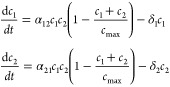
2where α_*ij*_ is the replication rate of species *c*_*j*_ in the presence of the mutualistic partner *c*_*j*_, *c*_max_ is the carrying capacity of the system, and δ_*i*_ is the death rate of population *c*_*i*_. As defined, we can see that no proliferation of any of the two partners will occur in the absence of the other due to the second-order kinetics that requires the product of the two concentrations (see Figure 2b).

### 2.3 Parasitism and Predator–Prey Interactions

Cooperators can outcompete other noncooperative species, but they are vulnerable to disruption by a cheater (a parasite). In this scenario, models predicted that spatial structure can help cooperators to defeat parasites,^30^ and this was shown experimentally using *E. coli* engineered communities.^16,29,31^ Cheaters can also have multiple effects (direct and indirect) on community dynamics. This is illustrated by a synthetic multispecies community, where the presence of fungi leads to bacterial parasitism on mammalian cells Weber et al.^32^

Here, *E. coli* is cocultured with transgenic human embryonic kidney (HEK) cells in a medium containing ampicillin, an antibiotic that restricts bacterial proliferation. HEK cells are genetically modified to produce an ampicillin hydrolase in response to high acetaldehyde levels. Consequently, when *S. cerevisiae*, a fungi which naturally converts glucose into acetaldehyde and ethanol, in present in close proximity to the coculture, HEK cells respond to the increase in airborne acetaldehyde by degrading ampicillin. With the decline in antibiotic concentration, *E. coli* starts proliferating at a much higher rate. This results in fast nutrient depletion, which consequently reduces growth and survival of HEK cells (see Figure 2d).

Qualitatively, parasitic interactions display an identical interaction network motif—a positive interaction in one direction and a negative one in the other direction—to predator–prey interactions. However, the temporal dynamics of these interactions can vary. Parasitic interactions may generally result in either stable coexistence or competitive exclusion, as illustrated in Figure 2d. In contrast, predator–prey systems can also display oscillatory behavior, as shown in Figure 2c.

The oscillatory pattern can be seen in the classical Lotka-Volterra model, defined in terms of the interactions between prey (*c*_1_) and predator (*c*_2_) populations using two coupled differential equations, namely:
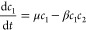
3for the prey, which grows at a rate μ and is predated at a rate β, and
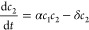
4for the predator, which grows at a rate α and decays with a rate δ. In nature, microbial predators have evolved unique strategies to grow and acquire their resources from other living bacterial cells.^33^ However, it is far from obvious how such an ecological motif can be implemented in a synthetic community.

The work by Balagaddé etal.^34^ solved the problem by finding a way to reproduce the *effective* positive nonlinearities that appear in the previous model. The study involved the manipulation of two distinct strains of *E. coli* to establish a controlled predator–prey dynamic within a confined environment using quorum sensing (QS), which enables density-dependent induction of genes across populations.^35^

In this case, prey cells consistently emit a QS compound. When this compound reaches high levels, predator cells generate an antitoxin. Conversely, predator cells continually release a distinct QS compound, which (at higher concentration) stimulates prey cells to produce a toxin, causing DNA damage and eventual cell death. Additionally, predator cells continuously produce the same toxin, but in this case, they can counteract it by inducing the expression of the antitoxin.

Given a population of prey cells *c*_1_ and a population of predator cells *c*_2_, the cyclic orbits can be reproduced by the following system of differential equations (adapted from the original system in Balagaddé et al.^34^Supporting Information):
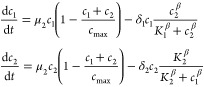
5where μ_*i*_ is the effective growth rate of population *c*_*i*_, *c*_max_ is the carrying capacity, δ_*i*_ is the maximum death rate, *K*_*i*_ is the concentration of the complementary strain at which death rate is half the maximum value, and β is the Hill’s coefficient of the interaction coupling the strains.

In contrast to the system represented by original eqs 3 and 4, an increased concentration of prey provides cross-protection, reducing the effective death rate of predators. However, predators are toxic to the preys. This creates a cyclic succession of populations: prey growth triggers antitoxin production in predators, allowing them to grow and replace the prey population by inducing toxin creation. As prey declines, predators start self-destructing as cross-protection diminishes, allowing prey to grow once again (see Figure 2c).

### 2.4 Environmental Self-Regulation

Ecological self-regulation is a fundamental concept in microbiology, encompassing the intricate mechanisms through which microbial communities maintain stability and balance within their ecosystems.^36^ Microorganisms continuously interact with each other and their environment, dynamically adjusting their populations and behaviors to sustain ecological equilibrium. These interactions shape community structure, function, and resilience, ultimately influencing ecosystem dynamics.^37^

One illustrative example of ecological self-regulation is the Daisyworld model,^38^ which serves as a simplified representation of a planetary ecosystem, where white and black daisies organisms regulate the planet’s temperature through their interactions with solar radiation.

Using synthetic biology, a possible implementation for a synthetic microbial Daisyworld was recently proposed by Maull et al.^39^ In this case, two cell populations growing in a chemostat, an acid-producer *c*_1_ and a base-producer *c*_2_, can self-regulate the environment. This regulation stems from inherent feedback mechanisms: acidification favors the growth of base producers, while alkalinization provides an advantage to acid producers. The equations that are used to describe the cell dynamics are a coupled set of equations describing the growth of each cell type, similar to standard replicator equations from population genetics:
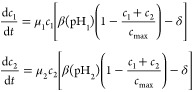
6where μ_*i*_ is the growth rate of population *c*_*i*_, *c*_max_ is the carrying capacity, and δ is the dilution rate. The function β describes the change in growth rate depending on the perceived pH,
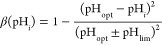
a symmetric, single-peaked function with its maximum located at the optimal growth pH, denoted as pH_opt_, and positivity maintained across the interval pH_opt_ ± pH_lim_. The perceived pH of each population deviates from the environmental pH, denoted pH_env_ due to the constant production of either an acid (*a*) or a base (*b*), slightly shifting the perception as follows:
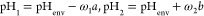
7where ω_*i*_ is the sensitivity of the population *c*_*i*_ to the produced compound.

Despite given an external input of either acid or base, pH_in_, the environmental pH is maintained close to the optimum by reorganizing the relative populations in a pH-dependent manner (see Figure 2e). The reorganization adjusts the production of acid and base, opposing the pH deviation due to the external perturbation.

Environmental self-regulation has also been studied within the context of B. subtilis biofilms, which have been shown to display pH self-regulation mechanisms to mitigate both internal (growth-related) and external pH challenges^40^ and within the context of bacterial pairwise interactions mediated through environmental modifications.^41^

### 2.5 Nontransitive Interactions

Game theory has long been employed in theoretical biology to investigate critical evolutionary phenomena such as the development of cooperative behavior, strategies in conflicts and fights, or interactions between hosts and parasites. Some synthetic counterparts of these systems have been obtained using small toxins (bacteriocines) synthesized by certain bacteria, toxic to other bacteria, and often closely related. Their production confers a competitive advantage to the producer by hindering the growth or even killing others.^42^ Toxin production typically entails some costs associated with synthesizing toxin and the lysis protein necessary for its release from the cell. Moreover, the producing bacteria are exposed to their own toxin, requiring a heightened immunity response. These emergent dynamics resemble the one shown by the classic “Rock-Scissors-Paper” (RSP) game.

This game was designed using three engineered populations:^43^ bacteriocin-producer cells (*c*_1_), sensitive cells (*c*_2_), and resistant cells (*c*_3_). The game can be represented, following Neumann and Schuster^44^ as
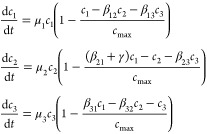
8where μ_*i*_ stands for the growth rate of population *c*_*i*_, β_*ij*_is the carrying capacity of the system and *c*_max_ is the cross-competition coefficient between populations. The parameter γ represents the disadvantage of the sensitive population *c*_2_ when competing with the toxin-producer population *c*_1_. RSP rules are obtained when *c*_2_ outcompetes the slower growing population *c*_3_, *c*_3_ outcompetes the slowest growing population *c*_1_, and *c*_1_ outcompetes the fastest growing population *c*_2_ due to toxin production. This results in orbits converging to a limit cycle, characterized by a cyclical succession of dominant populations where *c*_1_ replaces *c*_2_, then *c*_3_ replaces *c*_1_, and *c*_2_ replaces *c*_3_, thus starting the cycle anew (see Figure 2f).

These case studies capture the logic of ecological motifs, showing the potential for engineering them, but cannot capture the emergent patterns associated with whole communities. However, both *in vitro* and *in silico* paths can be followed to build and predict them.^45,46^ In the next sections, we address the problem of how synthetic biology can help design living communities and the lessons provided by looking at these problems on a multiscale approach.

## 3 Closed Ecospheres and Space-Life Support Systems

The previous section illustrates the potential of synthetic biology and mathematical modeling to design building blocks of ecological interactions. In all those examples, the environment where the population dynamics occur is implicit: limiting resources are somehow available. However, once we move away from small-scale cell cultures and bioreactors, we must face new challenges associated with multispecies communities and their environmental constraints. In this section, we consider a mesoscale level that includes a range of well-defined case studies where we aim to build an artificial community within well-defined boundaries. These ecosystems share a common goal: building self-sustained so-called *ecospheres* that display stable metabolic cycles and rich community compositions in analogy with the biosphere.

These systems have been studied since the 1960s as part of the engineering efforts toward space exploration and the engineering of so-called Life Support Systems (LSS),^47^ but also as a way to understand the requirements for a sustainable biosphere. In many cases, the difficulties and failures associated with their design have provided important lessons on how to predict their dynamical evolution.^48^ The potential for synthetic biology in harnessing these difficulties has shown that there is plenty of room for opportunities for addressing these problems while promoting novel microbial biotechnologies with broad implications.^49,50^

Early studies on materially closed but energetically open ecospheres revealed that self-sustained, metabolically active communities (including prokaryotes, eukaryotes and metazoa) could persist over decades.^51^ These closed ecosystems are, by definition, “synthetic”: the requirement of a boundary that forbids matter flows violates the open, out-of-equilibrium nature of natural ecosystems. Not surprisingly, the closed character of one such ecology imposes severe limitations on the space of viable ecospheres, given the strong ties between microbial growth and resource recycling. Quantitative methods and mathematical models have been key to providing bounds to the potential feasibility conditions for closed ecosystems,^52−54^ but only a few -and yet illuminating- experimental studies have been performed so far.^55,56^ One first estimation of the crucial conditions is sketched in Box 1 for a single species exploiting a single resource in a batch reactor. The general conclusion from this minimal model is that a critical boundary separates viable from nonviable life that balances microbial growth and recycling efficiency. Here comes one interesting and unexplored path to understanding and engineering closed ecospheres using synthetic biology: by assembling synthetic microbial communities,^57^ it would be possible to gain insight into the requirements for community-level complexity when matter flows are closed.^50^

Box 1 **Critical Thresholds for Synthetic Closed Ecosystems**

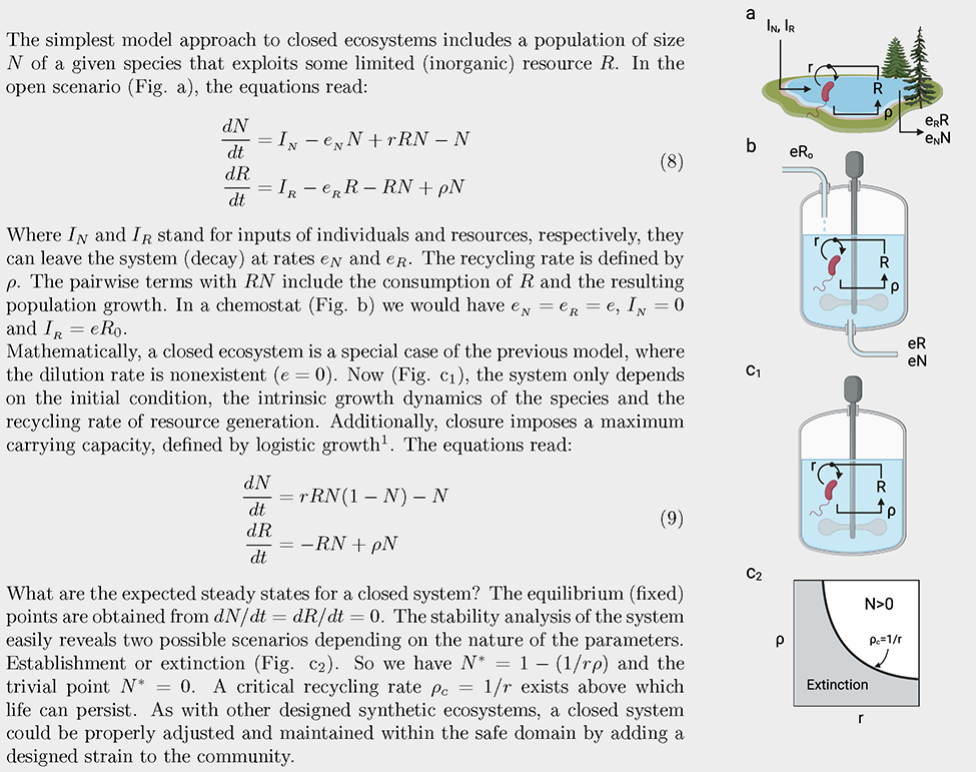

What can be learned from past and ongoing projects on LSS, and how can synthetic biology contribute to dealing with its limitations? The design of sustainable ecospheres for long-term space travel has been one of the main drivers for developing the science of closed ecosystems. It has inspired the emergence of a field, namely *space synthetic biology*.^58^ The most iconic early research examples are BIOS-3 and Biosphere 2. BIOS-3 marked one of the pioneering endeavors toward achieving full atmosphere closure to sustain two to three humans by cultivating higher plants, thus fulfilling human metabolic requirements. This closed ecological system operated for five months under human supervision.^59^ In contrast, Biosphere 2 encompassed an approximately 8000 m^2^ sealed structure housing a scaled-down replica of various ecosystems, including rainforest, desert, steppe, ocean, field, and farm. Its ultimate goal was to establish a dynamic equilibrium capable of self-sustaining the enclosed environment and a team of eight scientists.^60^

Any artificial closed environment, such as the ones outlined here, must have a set of reactions to perform cycles distributed among the species of the synthetic ecosystem interacting by metabolite exchange. These synthetic enclosed ecosystems must aim to zero matter loss, where all biochemical resources must be recyclable. In other words, the cycle of nutrients and matter must not be short-circuited by producing compounds that can not be biologically reprocessed. Microbial biotechnologies and synthetic biology will be pivotal in filling the gaps in the reaction cycles and harnessing available volatiles and waste resources. Methanogenesis and electrolysis, nitrification and denitrification or stress responses are important space synthetic biology processes that can allow the production of propellant, food, biopolymers, and pharmaceutical products. At the same time, they can be used in the waste recycling processes and life-support environmental control. Thus, increasing space exploration’s autonomy and long-term sustainability.^49,50^

**Figure 3:**
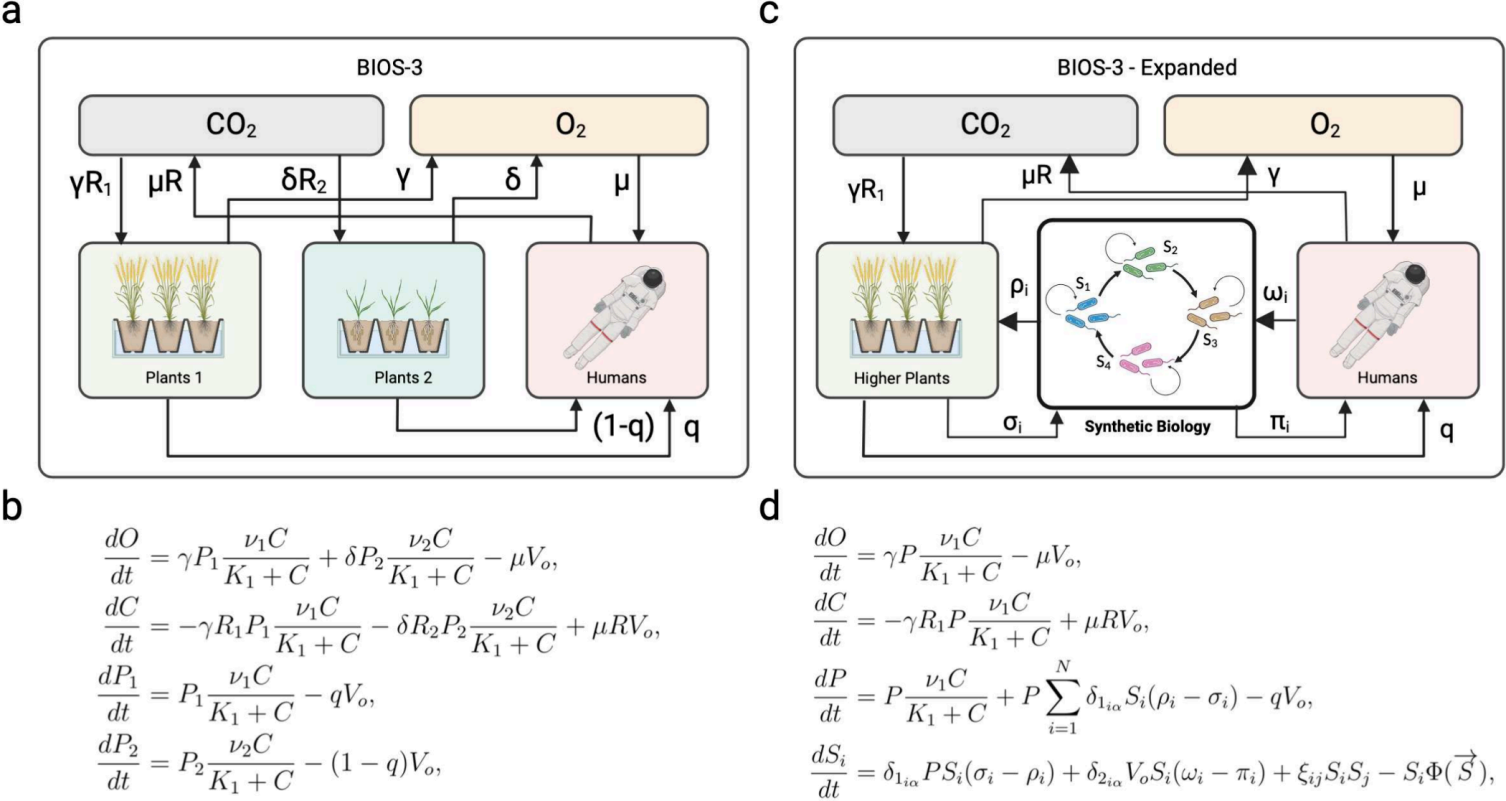
Systems and synthetic biology for life support systems. In (a), the BIOS-3 design of a closed ecosphere is represented here as a graph (including the closed cycling of O_2_ and CO_2_, along with higher plants and humans). This coarse-graining allows defining a set of equations (b) that describe the system.^48^ Here *O* = [O_2_], *C* = [CO_2_], and *P*_*k*_ indicate plant abundance; *V*_o_ is the rate of human metabolism; ν_1|2_ stands for plant maximum growth rate; γ and δ rate of plant growth; μ is rate of human metabolism related to oxygen consumption; *R*_1|2_ is the assimilation quotients for plants; *R* stands for human respiratory quotient and *q* the fraction of human metabolism supported by plants. Synthetic biology could enhance and stabilize this design, achieving a fully closed system and recycling plant and human waste as food and fertilizer are produced (c). Here, an extra compartment is added. The tag “Synthetic Biology” stands for the necessary closure of metabolic pathways, here to be conducted in bioreactor tanks. The corresponding mathematical model (d) is obtained from (b) by adding *S*_*i*_, where *i* = 1, ..., *N*, where *N* is the number of species performing biochemical reactions. Here we could propose a closed catalytic architecture, i.e., hypercycle dynamics governing *S*_*i*_. Here δ_1_*iα*__ and δ_2_*iα*__ stand for Dirac’s delta function to tag who is producing or recycling from or to plant and human compartment, respectively; i.e., δ_*iα*_ = 1 when *i* = α and zero otherwise. The catalytically assisted replication species *j* provides to *i* is denoted by ξ_*ij*_. Recycling and production rates are ω_*i*_, σ_*i*_ and π_*i*_, ρ_*i*_, respectively. The last term, Φ(*S*), is the dilution outflow.

The pioneering design of BIOS-3 illuminates the basic principles and how quantitative dynamical models can be formulated. In Figure 3a-b, we summarize the connection between biotic and abiotic components and a minimal model based on four key variables. Here, four variables capture the systems-level features, namely: (a) two key abiotic components (oxygen and carbon dioxide) and (b) two types of plants that regulate the closed environment. Here, the human factor is encapsulated in the human metabolic rate *V*_*o*_, excluding them from the mass conservation laws. Therefore, this model is completely closed regarding the atmosphere.^a^

How could we modify the previously designed flows using synthetic biology? Again, quantitative population models can give us insight. The mathematical modeling of BIOS-3 (Figure 3b) reveals the presence of an equilibrium point but also that this point is unstable under small disturbances.^48^ This implies that some external intervention to tune the parameters is a necessary requirement for system-level robustness. However, one interesting prediction from the synthetic environmental self-regulation (ENV regulation in Figure 2) might provide an alternative. The prediction^39^ is that a diverse community (instead of simply two engineered strains) is much more likely to achieve environmental self-regulation. A full closure would be achievable with the addition of a rich synthetic community necessary to close the matter fluxes. Such a compartment (tagged as “synthetic biology” in Figure 3c) would allow the recycling of human and plant waste by a network of microorganisms that could be hybrid, i. e. including both natural or equipped with designed *de novo* capabilities. Here, the reactions necessary for achieving complete closure, considered subcompartments, would be represented by formulating differential equations for *N* strains *S*_*i*_, with *i* = 1, ...*N*, where *N* denotes the total number of species involved in biochemical reactions. Remarkably, ongoing for over 30 years, there has been an endeavor to design and construct a hierarchically controlled ecological life support system facility, using microorganisms aimed at recovering resources from waste, known by the acronym MELiSSA.^61,62^ Alternatively, The path toward LSS and ecospheres will require the formulation of ecological motifs that can cope with the potential outcomes of engineering designs, ensuring ecological resilience, long-term persistence and reliability. Network structures, like producer-consumer-reducer chains, could close a given system, where one species could reduce a given substrate to feed the next.^63^ One key approximation would be the exploitation of hypercycles;^64^ where all species involved in the ecosystem are entangled in a closed catalytic architecture, ensuring this way their persistence, see Figure 3d. As discussed in^64^ within the context of synthetic biology, the mutual dependencies between the members of the hypercycle (indicated by the terms ξ_*ij*_*S*_*i*_S_*j*_ in Figure 3d) guarantee the presence of all the components. By distributing a given number of functionalities over different strains, their persistence would guarantee their functional contribution to the system’s performance. Such a synthetic multispecies community could be engineered using the appropriate syntrophic interactions.^65^

Finally, we could wonder which are the candidates for such engineered communities? Particularly outstanding ones are cyanobacteria. Along with microalgae, they have been proposed as key components for photobioreactors.^66^ Desert cyanobacteria, particularly those adapted to extreme environments, have been shown to be highly resistant to the harsh conditions of space and Martian simulated conditions.^67^ Enhancement of these strains would be obtained using synthetic biology and not only allow for oxygen production but also to obtain energy, food, fuel and a variety of materials.^68^ Their relevance will be highlighted when discussing synthetic ecosystems on the largest scales, as discussed in the next section.

## 4 Synthetic Biospheres

Our previous examples reveal the potential for interrogating the nature of ecological interactions by implementing micro- and mesoscale synthetic systems and their associated mathematical descriptions. They can also provide valuable insights into our understanding of planetary boundaries and novel ways of thinking about the future of our biosphere. The environmental regulation motif (Figure 2) offers a unique opportunity for small-scale experimental testing of questions regarding Earth systems science. In particular, it would help understand the interplay between microbial community responses to externally changing environments and how feedback loops can generate homeostasis.

In the previous section, we have also touched on this problem as we considered the limitations imposed by how a sustainable closed ecosystem would allow for persistent communities. In both cases, systems and synthetic biology approaches shed light on potential conditions for ecological persistence. Here, mathematical models are helpful to define the causal connections between different variables and to predict the likelihood of success for a given engineered design. The feasibility domains obtained from these models also provide a no less critical feature: their boundaries define the conditions for tipping points. Can this conceptual framework address the ongoing biodiversity and climate crisis?

The existence of tipping points in natural ecosystems was early raised within the ecological literature^69^ and has become a significant research area in the past decades.^70^ Due to diverse effects of Anthropogenic origin (global warming, contamination, habitat loss and intensive agriculture), many extant ecosystems face the possibility of rapid degradation as key environmental parameters cross critical thresholds.^71,72^ Far from happening in the distant future, these events will likely become a reality over the next decades.^73^ Along with much-needed policies regarding conservation, sustainable growth and the transition toward green energy sources, it has also been proposed that synthetic biology could play a relevant role^20−22,74^ as an alternative to geoengineering scenarios. Because these bioengineering strategies require the inoculation of engineered microorganisms in extant habitats, this *terraformation* approach is an intervention scenario that could give rise to new communities. This path toward the use of synthetic biology to modify ecosystems has been gaining momentum, and microbial biotechnology is now a largely unexplored but up-and-coming alternative that has been proposed within the context of bioremediation,^23^ dryland preservation^75^ or carbon sequestration.^76^

One particular case study involves arid and semiarid ecosystems. These so-called Drylands cover around 45% of emerged lands and host more than one-third of the human population, and they are expanding while being threatened by global warming.^77,78^ A very important contribution to dryland stability and resilience is the soil microbiome.^79−81^ In this context, some particular species, such as cyanobacteria, are known to play a key role and have been used in restoration efforts where their inoculation can enhance carbon sequestration.^82,83^ Not surprisingly, the potential for synthetic biology as an enhancer of this process has also been suggested.^75^

Along with field studies (sometimes hand in hand), mathematical models have successfully addressed the problem of the origin of alternative states and catastrophic shifts.^84^ One example is the so-called green-desert transition, where aridity values larger than *a* = 0.8 trigger a shift from a vegetated state to a desert-like state. Recent studies on global drylands have shown that several regime shifts seem to be involved in the response of drylands to aridity levels.^78^ We can illustrate this phenomenon using a toy model that captures some key components responsible for these shifts, where the population cover of a given area is indicated as *x* and modeled as a nonlinear equation:
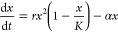
9*K* is the carrying capacity, while *r* and α are plants’ growth and death rates, respectively. Notice a quadratic term in the growth function characteristic of systems that exhibit *facilitation*: plants enhance the growth of other plants, usually mediated by soils. Because of this nonlinear term, the response of this system to external stress, quantified by α, is abrupt.^b^ Specifically, if α < α_*c*_ = *r*/4, a stable vegetation cover will be observed but will vanish for α > α_*c*_. It can be shown that the system shifts from a state *x* = *K*/2 (thus displaying a half-covered area) to the desert (x* = 0) state.

Dedicated quantitative and theoretical efforts have revealed that collapse is a likely future scenario for facilitation-driven communities, such as drylands.^84^ Can such undesirable shifts be avoided or at least prevented? Are there potential ways of reducing the impact of aridification or even halting these systems in a safe state, avoiding catastrophic transitions? Is there room for synthetic biology as a way to stop ecosystem shifts? The answer to these questions requires considering the problem of multiple ecological scales.^23^

In this *terraformation scheme*, synthetic strains allow us to bypass an obvious limitation of most geoengineering designs: the ”machines” do not need to be built since each engineered cell can replicate itself. This advantage from biology makes it possible to spread on a given scale.^21^ This, of course, raises several issues regarding safety:^19,85^ can these interventions have “unintended consequences”? Interestingly, the answer to this question must be found in the collective behavior of ecological networks. Under this view^39,86^ engineering is designed to enhance biodiversity, and biodiversity defines the conditions for containment.

This engineering departs from the standard, top-down picture aimed at predictable control over the system properties. Instead, the terraformation scheme is an *emergent engineering* approach that follows two essential design principles. First, a resident community member is chosen as the candidate species to be modified.^22^ The best choice here would be some species of cyanobacteria, which have a well-known impact in dryland ecosystems. More importantly, they are *ecosystem engineers*: they have a specially disproportionate impact on the flows of matter and energy of the dryland soils. The term is especially relevant within ecology^89^ and provides a helpful guide to identifying keystone species to define restoration strategies.^90^ In other words, *we define a terraformation strategy grounded in engineering ecosystem engineers*.

The reason for choosing a microbial species from the resident community is that, as a member of a coevolved ecological network, we expect this species to display some stability.^86^ Once the candidate has been isolated and modified, it is returned to the original community equipped with a given, designed functionality while preserving the wild-type interactions with other species.

The second part of this approach is based on a systems-level property strongly tied to ecosystem health: biodiversity,^87^ which plays a leading role (along with soil organic carbon) in preserving drylands.^91^ While most studies within synthetic biology have addressed the problem of containment by actively pursuing gene-level control of growth,^92^ here we suggest that biodiversity, as well-known from invasion ecology studies,^93,94^ will act as a firewall.

**Figure 4:**
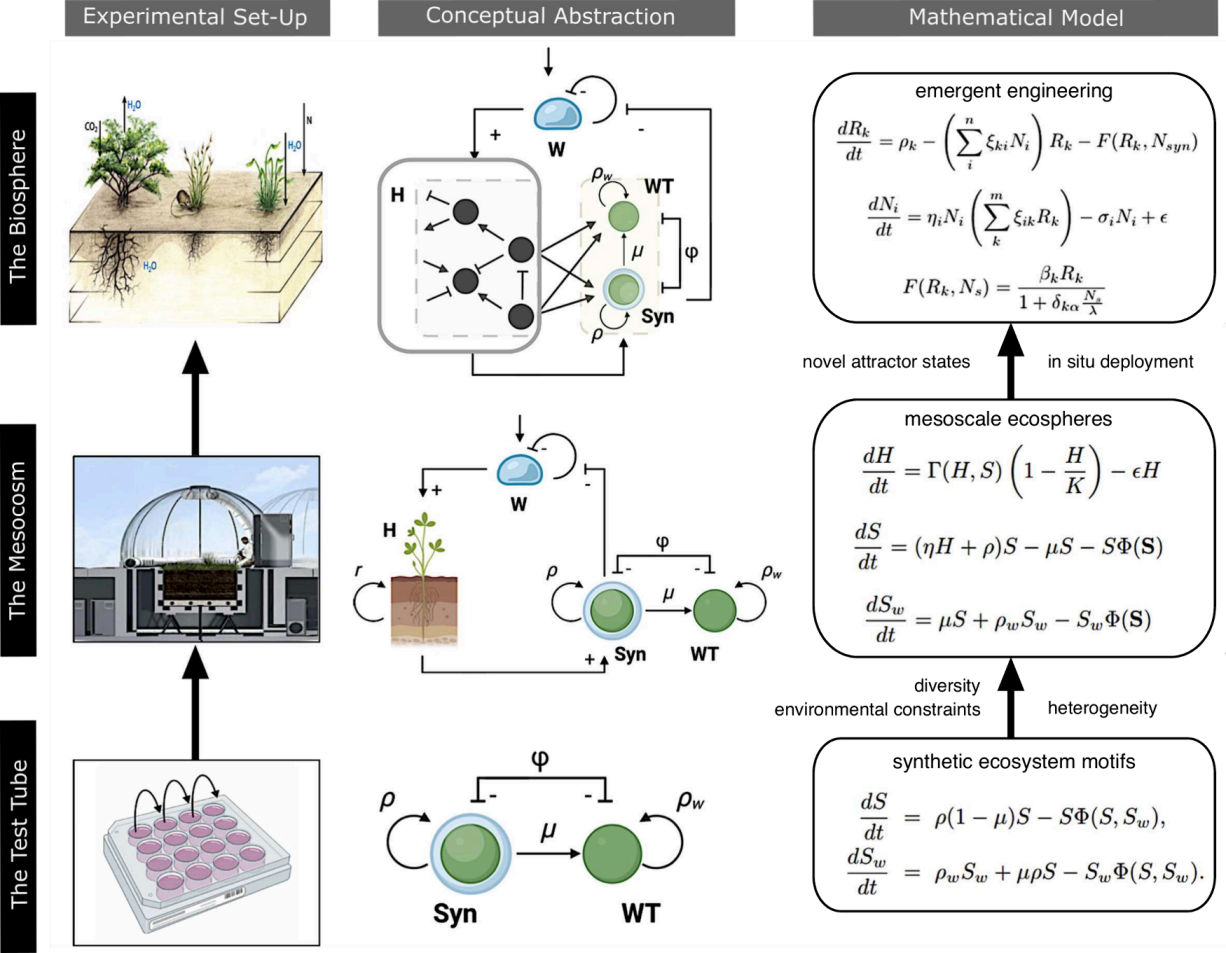
Synthetic ecosystems for dryland restoration and conservation. A visual summary of the three levels of analysis. From bottom to top: (1) competitive strains in liquid media, theoretically described by standard ode models, with a ”test tube” implementation; (2) micro/mesocosms implementation of engineered cooperators and the soil crust, where models are now extended to include environmental feedbacks; and (3) large-scale systems can be approached using formal models involving a network-level description. As we move from one level to another, new, emergent properties must be considered.

What kind of extra functionality can be used? An example of drylands would be a genetic construct that helps fight aridity by reducing water losses. Once again, cyanobacteria are known to secrete extracellular polysaccharides that contribute to soil health in different ways, from the increase in organic carbon to the improvement of soil stability and water retention. Water losses could be reduced by engineering a strain to produce a more efficient extracellular molecule, and the possibility of an undesirable ecosystem shift could be prevented (Box 2). As shown in,^87^ the population-level impact on the rest of the community is typically negligible. All species remain basically in the same state, with very rare extinctions and, in some cases, showing augmentation. This is the first evidence that the risks associated with engineering in the wild employing synthetic biology might have been overestimated. As the population model suggests, the potential unintended consequences might be negligible under our emergent engineering approach. Here, experiments in micro- and mesocosms under controlled conditions will be required to test the validity of the model approximations and its predictions. In this context, the lessons provided by small- and medium-scale engineered communities (the two lower layers in Figure 4) and those from the fields of invasion ecology can inform us about the feasibility of these interventions.

Box 2 **Emergent Bioengineering: Systems Biology of Ecosystem Terraformation**

What can be expected if we inoculate a given synthetic strain in a community? Mathematical models help predict the outcome of this intervention and the role played by biodiversity. One general scenario involves a set of species , namely, Ni where *i* = 1, 2, ... *n*, and a set of resources {*R*_*k*_}, where *k* = 1, 2, ... *m*. The growth rates for each resource and species are represented by ρ ∈ (0, 1) and η ∈ (0, 1), respectively.^87^ One is a strain (*N*_syn_) engineered from a resident species, thus sharing all the ecological interactions with the wild type. This synthetic strain can reduce the loss of a given, shared resource. The equations read:
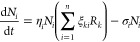
11for the species populations, whereas the resources will change in the following:
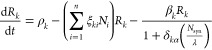
12Whereas most of the previous set of equations corresponds to the standard resource-consumer models, the last term in eq 12 introduces the effect of the synthetic strain (*N*_syn_). As we can see, it has an inhibitory effect on the loss of the α-th resource (here δ_*k*α_ = 1 if *k* = α and zero otherwise), whereas in the absence of the synthetic strain (i.e., if *N*_syn_ = 0), *R_k_* decays at a rate β*k*. By modeling these populations, it has been shown that, under the proposed engineering scheme, biodiversity acts as a firewall for expanding the synthetic strain, which performs its function and improves the community without species loss. Because of this shared resource’s effective decay, there is an emergent community-level readjustment: we do not intend to predict (as in a top-down scenario) how different species will behave. Instead, the fact that biodiversity enhances ecosystem functionality as an indicator of health makes it the target of the intervention.

## 5 Discussion

Understanding natural ecosystems, repairing dysbiotic microbiomes, planning for future space missions, and even engineering our biosphere are examples of the wide range of problems where synthetic biology can play a role within ecosystems across scales. This paper synthesizes ideas and case studies concerned with ecological communities that are fully designed using genetic engineering or built as hybrid assemblies. This includes fully engineered microcosms, augmented ecospheres or terraformed ecosystems. A common thread in our analysis involves using mathematical models that help us understand the implications of nonlinear interactions and predict potential outcomes. This systems biology view connects with the traditional quantitative study of ecological assemblies through population dynamics.

**Figure 5:**
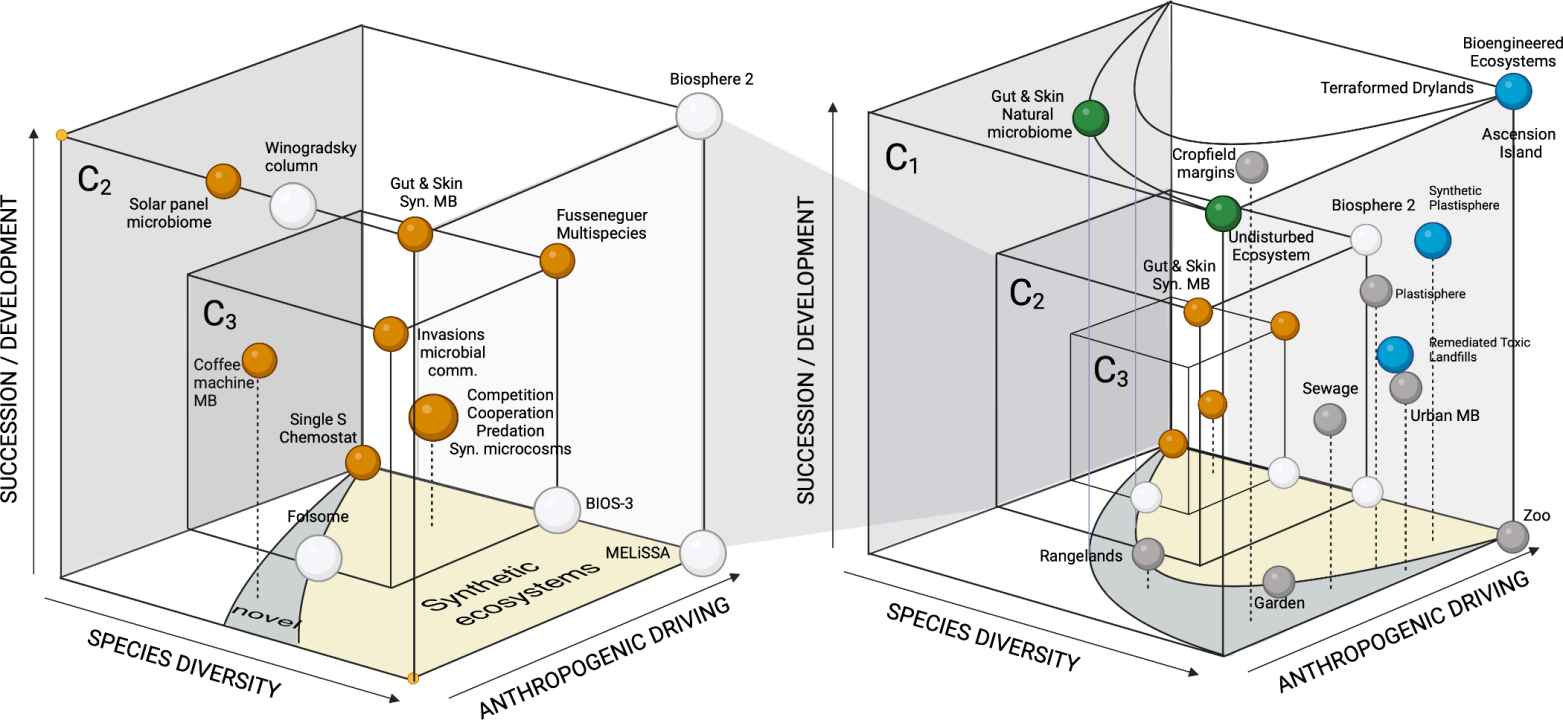
Synthetic ecosystems in context. A space is defined using three axes, namely, the degree of community development, species (strain) diversity, and the amount of human intervention. Each sphere represents a given system and locations are just relative to each other, and the three scales considered in this paper appear as three nested cubes (*C*_1_ ⊂ *C*_2_ ⊂ *C*_3_). Novel and synthetic domains are indicated by the gray and yellow areas, respectively. Anthropogenic actions create conditions for the emergence of so-called *novel ecosystems*.^88^ Beyond, *synthetic ecosystems* span out of design effort. On the left, we have the test tube scale designs that involve standard motifs, small-scale microbiomes living in man-made artifacts, mesocosm experiments and life support systems. These two scales are shown within the larger cube *C*_1_ (right) that contains undisturbed and designed (terraformed) ecosystems as two corners on the right upper part. Gray spheres stand for human-driven ecosystems and blue marbles indicate scenarios of ecosystem intervention.

Although each scale is valuable in itself, they are by no means disconnected. Fundamental dynamical and structural properties are crucial to predict the expected outcomes of some types of interactions within more complex communities. Simple regulatory feedbacks provide evidence for potential designs of microbial assemblies that could offer enhanced resilience to closed or semiclosed ecospheres. The lessons learned from hybrid mesocosms could be instrumental in future scenarios based on our terraformation scheme. This rich range of designs and scales is summarized in Figure 5 using the concept of a *morphospace*.^95^ In a nutshell, three axes involve (1) the degree of human intervention (from no intervention to complete engineering), (2) species/strain diversity and (3) the role of development in generating the outcome.

Here, each case study is represented by a sphere and their location is defined relative to others (and thus, there is no metric). We use two highlighted areas indicating synthetic ecosystems and the so-called *novel ecosystems*. The latter is associated with losing valuable, historical habitats. These include communities that result from nonhistorical networks of species arising from human-disturbed habitats, environmental change or species invasions. The whole spectrum of possibilities is shown in the right cube (*C*_1_) cube, which encapsulates both microscale (*C*_3_) and the mesoscale ecospheres (*C*_2_). On the left we zoom out the smaller cubes, dominated by synthetic ecosystems. As we can see, small-scale systems occupy a domain of the space that would be otherwise empty.

As we have seen in the previous sections, the relevance of synthetic biology percolates across scales. The study and modeling of synthetic ecosystems can be extended into multiple directions, each addressing relevant questions. Here, we provide a list of potential paths for exploration:

1 Although the microbial consortia described above follow the logic of ecological interactions in nature, this constraint does not necessarily limit the potential design principles, particularly when engineering computations with living cells.^96^ In particular, it has been shown that complex functionalities can be built on engineered consortia that do not follow either biological or standard engineering designs.^97^ What are the corresponding motifs and the alternative designs for ecological engineering?
2 Who needs to be targeted when modifying ecospheres or natural communities? Considering single-species changes is one particular scenario within engineering complex communities.^98^ Here a crucial problem is dealing with the balances between competition and cooperation and the detrimental role played by mutants.^99^ Notably, despite the success of microbiome transplants, little is known regarding theoretical models about how these transplants work. Developing synthetic models to address this problem would benefit biomedical research and new ecosystem bioengineering strategies.
3 Detailed quantitative network models require estimates about the sign and weight of species–species interactions. This is a challenging problem that has received much attention, and mathematical models involving using generalized Lotka-Volterra equations, namely:
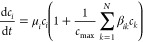
10Saavedra et al.^100^ used for example in the study of the gut microbiome.^101^ The challenge here is to determine the matrix *B* = (β_*ij*_) that captures the strength of competitive interactions and influences community resilience and responses to engineering. Recent work on community transitions^102^ provides a helpful estimate of these properties (based on an entropy measure) that could be used as a quantitative approach to determine the likelihood of microbiome shifts.
4 Engineering efforts affecting extant communities, such as drylands, present a significant challenge regarding the interactions between scales. The microbiome affects soil carbon content, which influences plant cover, and the quality of the plant community influences soil properties. On the other hand, we are considering changes that can percolate across whole landscapes, which can display several sources of heterogeneity. In this context, future efforts might benefit from machine learning techniques that can provide a system-level integration of data-driven information about responses across scales. Future models of terraformation should address this multiscale picture.
5 In our previous examples, we assumed that synthetic biology has microbial candidates as potential chassis for engineered designs, and the population dynamics occur at the bacterial-resource scale. Viruses, on the other hand, and despite their tiny contribution to biomass, are acknowledged as crucial players in the dynamics of microbiomes, and they are understudied.^103^ The impact of viruses and their potential role as vectors for ecosystem engineering should be explored using mathematical models that introduce the virome in an explicit manner.
6 Under an evolutionary perspective, it has been suggested that synthetic ecosystems can shed light on a variety of open problems regarding the evolution of complexity beyond the species level.^104^ Here, too, ongoing efforts to build and augment in vitro synthetic interaction networks,^105^ along with new population dynamics models, could help define scenarios of ecosystem transitions and the role played by contingency versus robust network properties on the tempo and mode of microbiome evolution.

The ecological perspective across various life sciences fields, from cancer research to biomedicine, underscores the value of studying synthetic ecosystems as a critical link between models and reality. Building on the tradition of creating microcosms as partial yet representative in vitro simulations of natural communities, synthetic biology offers a unique framework that integrates experimental studies with mathematical models. This integration facilitates connections across different scales and conceptual frameworks, linking areas as diverse as invasion ecology and metabolomics. Insights from engineering approaches to the gut microbiome^106^ and its modeling^101,107,108^ can inform the use of synthetic biology in restoration and conservation efforts. In contrast, the systems perspective from these efforts can enhance our understanding of organismal-level ecology. In all these contexts, theoretical models are expected to play a crucial role in establishing unifying principles.

## Footnotes

a A closed microbial system confined in a container would easily impose a carrying capacity due to space constraints, i.e., proximity-dependent inhibition. Conversely, an unbounded scenario would present a much more restrictive solution, being 1 = *rρ* the only condition for long-term *N** > 0.

b This is a simple mathematical model of the Allee effect, where a population will thrive, provided that minimal population size is achieved. This is obtained here by first finding the fixed points of the dynamics, i.e., the *x**, such that d*x*/d*t* = 0. In this case, one solution is the extinction (desert) state *x** = 0, whereas two extra states *x*_±_ are found by solving the quadratic equation *x*^2^ – *Kx* + *αK*/*r* = 0. The solutions of this equation exist only if 1 – 4α/*r* ≥ 0, which gives the critical condition.
